# Handgrip strength thresholds associated with metabolic syndrome risk in children and adolescents: a systematic review and meta-analysis

**DOI:** 10.4178/epih.e2024047

**Published:** 2024-04-24

**Authors:** Hye Ah Lee, Seunghee Jun, Hyesook Park

**Affiliations:** 1 Clinical Trial Center, Ewha Womans University Mokdong Hospital, Seoul, Korea; 2 Department of Preventive Medicine, Ewha Womans University College of Medicine, Seoul, Korea; 3 Graduate Program in System Health Science and Engineering, Ewha Womans University College of Medicine, Seoul, Korea

**Keywords:** Adolescent, Children, Handgrip strength, Metabolic syndrome, Meta-analysis, Systematic review

## Abstract

**OBJECTIVES:**

Certain studies have reported that handgrip strength (HGS) is associated with metabolic health risks in children and adolescents, and some studies have suggested HGS thresholds for identifying poor metabolic health. Therefore, we aimed to determine the HGS thresholds associated with metabolic syndrome (MetS) in children and adolescents through a systematic review.

**METHODS:**

We searched 3 electronic databases from their inception until October 2023 to identify original papers that focused on children and adolescents and assessed their risks of MetS according to specific HGS values. Studies were selected for inclusion through a planned screening process based on specific criteria. The Quality Assessment Tool for Diagnostic Accuracy Studies version 2 (QUADAS-2) was used to evaluate quality, and a meta-analysis was performed using the diagmeta R package to suggest the optimal thresholds.

**RESULTS:**

From the search, 8 studies were selected for this systematic review. For detecting MetS risk, the optimal threshold for HGS (defined as relative HGS by adjusting for body mass) was found to be 0.422, with a sensitivity of 76.7% (95% confidence interval [CI], 64.0 to 85.8) and a specificity of 62.9% (95% CI, 56.9 to 68.5). The stratification analysis by sex resulted in optimal thresholds of 0.416 for boys and 0.376 for girls. Additionally, when the data were stratified by age, the thresholds were 0.356 for children and 0.416 for adolescents.

**CONCLUSIONS:**

Our results provide practical information for detecting high-risk groups and encouraging strength-related activities that may reduce the risk of MetS in children and adolescents.

## GRAPHICAL ABSTRACT

[Fig f5-epih-46-e2024047]

## Key Message

• This study conducted a systematic review and meta-analysis to propose the optimal handgrip strength (HGS) threshold for detecting the risk of metabolic syndrome in children and adolescents.

• The results provide practical information for primary clinical and public healthcare, as they can be used to identify high-risk groups in need of strength-related interventions to reduce the risk of metabolic syndrome in children and adolescents.

## INTRODUCTION

As a secondary effect of the coronavirus disease 2019 (COVID-19) pandemic, a clear increase has occurred in the prevalence of obesity in children and adolescents [1]. Several studies have consistently noted a steeper increase in prevalence in younger children [2-4]. As obesity increases, the poor metabolic health of children and adolescents can naturally be expected to increase. Metabolic syndrome (MetS) is a clustering of cardiometabolic risks that may be observed even at a young age. According to a recent meta-analysis of 550,405 children and adolescents in 44 countries which used Cook’s modified criteria, the prevalence of MetS in children and adolescents as of 2020 was estimated to be 2.8% and 4.8%, respectively, corresponding to 25.8 million children and 35.5 million adolescents [5]. The childhood onset of disease susceptibility is a precursor for various non-communicable diseases later in life; therefore, identifying effective preventive measures is urgent.

Several studies have reported that handgrip strength (HGS) as a muscle strength indicator is related to MetS in children and adolescents, and some studies have proposed assessing MetS risk based on optimal thresholds for HGS measurements [6-8]. These studies have presented thresholds that vary by sex and age. Objective thresholds can be used to identify individuals at high risk of poor health based on fitness test results and may reduce confusion in communication. Additionally, several systematic reviews and meta-analyses have been undertaken to comprehensively describe muscle strength and metabolic health risks. A review by de Lima et al. [9] of 13 studies evaluated muscle strength and MetS in children and adolescents. Another systematic review looked for an association between musculoskeletal fitness and MetS in children and adolescents and conducted a meta-analysis of the discrimination power of musculoskeletal fitness to detect MetS [10]. However, no study has comprehensively summarized and proposed optimal thresholds for screening HGS as a predictor of MetS in a screening setting.

Therefore, through a meta-analysis, this study aimed to quantitatively present the accuracy of HGS values for determining the potential risk of MetS in children and adolescents and to suggest optimal HGS thresholds.

## MATERIALS AND METHODS

### Search strategy

We identified relevant studies by searching electronic databases (PubMed, Web of Science, and Scopus) for original work published up to October 2023. The focus was on studies related to HGS and MetS risk, and the search terms were chosen by referring to terms used in existing studies [9-11]. Although we expanded the search to include studies referenced by systematic reviews, no additional studies were found. Regarding the date of publication, no start date was imposed for the database searches. However, the scope of inclusion was restricted to studies published in English. Supplementary Material 1 provides the search terms in detail.

### Eligibility criteria

Studies were considered eligible if they met the following criteria:

(1) Because no consensus exists regarding the definition of MetS in children and adolescents and its prevalence is low, the concept of a continuous metabolic syndrome score (cMetS) has been accepted by some researchers. In those cases, MetS risk was determined by the study’s cMetS value and included if it could be clearly expressed as a binary variable. Studies were also included if they defined their parameters using existing criteria for MetS in children and adolescents (e.g., International Diabetes Federation [IDF] classification, Cook criteria, Ferranti criteria); (2) Studies assessing the association between MetS and HGS levels were included when HGS levels were defined with enough specificity to construct a 2×2 table for MetS diagnosis by HGS; (3) Studies focusing on research subjects under the age of 18 years were included; (4) Observational studies with a scope that was not limited to specific populations (e.g., athletes and patients with specific diseases) were included.

The following categories of studies were excluded:

(1) Types of publications such as reviews, conference abstracts, and letters; (2) Studies that lacked explicit threshold values for HGS or did not assess MetS risk in a dichotomous manner; (3) Studies that measured HGS but evaluated it in combination with other indicators.

### Study selection and data extraction

All eligible records from the databases were downloaded and then uploaded into Rayyan, a tool for systematic review [12], where the records were checked for duplication. Screening of titles and abstracts against the eligibility criteria was carried out independently by 2 authors (HAL and SJ). This was followed by full-text screening, which required consensus for final inclusion. Any discrepancies were resolved through discussion. A meta-analysis of multiple thresholds allows for multiple thresholds in 1 study. Therefore, when studies with the same data source were conducted, the proposed thresholds were different, and the inclusion criteria were met, those studies were selected for the current review.

The primary parameters of interest were the HGS thresholds and their corresponding diagnostic accuracy indices. For the selected studies, we extracted specific descriptive data including the author(s), year of publication, country, study design, participants’ sex and age, sample size, prevalence of MetS, definition of MetS, HGS measurements, HGS thresholds, and diagnostic accuracy indices (e.g., sensitivity and specificity). To generate multiple thresholds summary receiver operating characteristic (mtsROC) curves, each HGS threshold and its corresponding numbers of true positives, true negatives, false positives, and false negatives were recorded. In cases where the exact number of participants was not presented, we estimated the frequency using the available information. Additionally, when necessary, we requested further information from the papers’ corresponding authors via email.

### Assessment of risk of bias

The risk of bias in each study was assessed using the Quality Assessment Tool for Diagnostic Accuracy Studies version 2 (QUADAS-2) [13]. The tool consists of 4 domains: patient selection, index test, reference standard, and flow and timing. Based on the signaling questions, each domain was assessed as “low,” “high,” or “unclear.” If a given study had high scores on 1 or more signaling questions in a domain, that domain was rated as having a high risk of bias [14]. The results of the analysis of bias were displayed visually using the robvis R package.

### Statistical analysis

In summarizing the diagnostic accuracy of HGS for detecting MetS risk, we employed a random-effects bivariate model based on 18 parameters. For the current analysis, allowing for a single pair of sensitivity and specificity, when multiple thresholds were presented per study, we selected one threshold per sex, age group, and study, based on the maximum value of Youden’s index. Among studies from Korea using data from the Korea National Health and Nutrition Examination Survey (KNHANES), 1 study that presented results by age group was selected to avoid duplication. Among the 4 studies conducted in Korea, Lee et al. utilized data spanning from 2014 to 2017, while the remaining studies covered the period from 2014 to 2018. In the studies by Jung et al. and Choi, participants were excluded based on the fasting time before blood collection, and in the study of Jung et al., participants with inappropriate fasting glucose values were additionally excluded. Meanwhile, unlike the other studies in Korea, Ko et al. applied the MetS criteria proposed by Ko et al. applied Cook’s criteria to define MetS risk (Table 1) [15-18]. The characteristics of the included studies were presented in terms of sensitivity, specificity, positive likelihood ratios (LRs), negative LRs, and diagnostic odds ratios (DORs), with 95% confidence intervals (CIs). The results of the bivariate model are presented in the current study as summary values of sensitivity, specificity, and area under the curve (AUC). We also quantified between-study heterogeneity using *I*^2^ statistics, following the Zhou and Dendukuri approach. Additionally, our analyses were stratified by sex and age group.

To identify optimal thresholds for MetS risk detection using HGS, we extracted an additional 14 thresholds beyond the 18 used in the bivariate model. Consequently, a total of 32 different thresholds from 8 studies were analyzed using a multiple thresholds model with the diagmeta R package. To reduce heterogeneity between studies, if the HGS threshold was presented as the sum of values from both hands, the value was divided by 2 for use in our analysis. The optimal threshold was defined as the point at which the maximum value of the weighted sum of the sensitivity and specificity values was obtained. As the thresholds increased and metabolic risk decreased, we multiplied the thresholds by -1. It can be modeled as one of 8 mixed linear models. For the meta-analysis, the model fits the data with all available thresholds across all studies. The best-fit model was selected following the recommended methods [19]. The results are presented as mtsROC curves, optimal threshold, and the sensitivity and specificity for that threshold.

As a subgroup analysis, we classified the age groups into children and adolescents, based on whether the average age was below 10 years of age (with 6 datasets for children and 26 for adolescents). Further analysis was conducted by sex, with 16 datasets each for boys and girls. A sensitivity analysis was also conducted, which considered heterogeneity depending on the measurement tools.

### Ethics statement

This study employs a meta-analysis research methodology, using data from previously published articles. Since this study did not involve human subjects, institutional review board approval was not required.

## RESULTS

**Figure 1.**
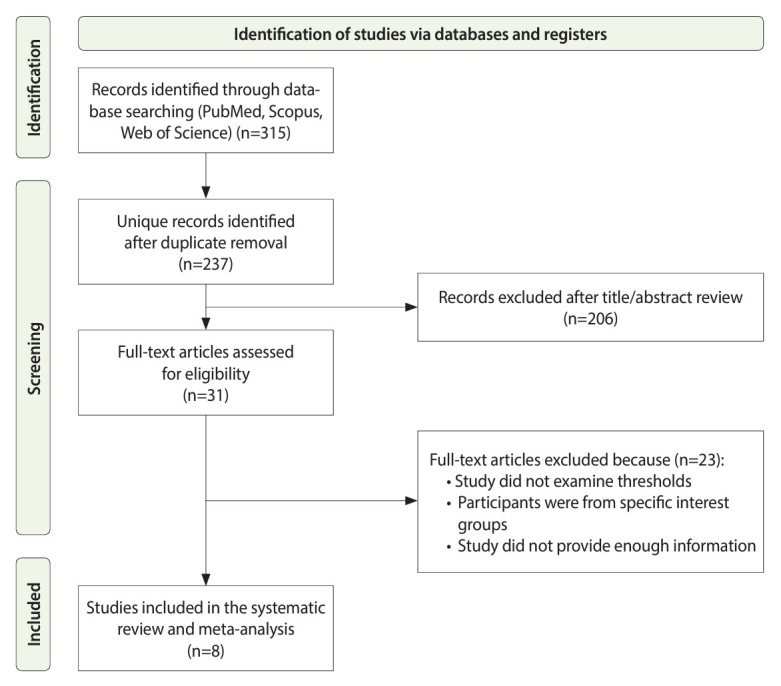
Flowchart of the literature search and selection process.

The study selection flow diagram is shown in Figure 1. A total of 315 studies were identified through database searches. Among these, 78 duplicate studies were excluded, followed by a screening of titles and abstracts. Subsequently, 23 studies were excluded after full-text review, resulting in the final inclusion of 8 studies [7,8,15-18,20,21] in this study.

Evaluation using QUADAS-2 showed low levels of bias across the studies. Regarding the risk of bias, because of the lack of a standard definition for MetS in children and adolescents, all studies were rated as unclear for the question, “Is the reference standard likely to classify the target condition correctly?” Furthermore, 2 studies [8,20] did not use random sampling to recruit participants (Supplementary Material 2).

All 8 selected studies were published in English between 2016 and 2022. Among them, 4 studies [15-18] were conducted in Korea using data from the KNHANES. In all studies, HGS was assessed as relative HGS by adjusting for body mass. One study conducted in the United States [7] assessed HGS for the dominant hand, while 2 studies [16,18] evaluated the sum of the maximum values of HGS for each hand. One of these studies used hydraulic dynamometers, and the other utilized spring-type dynamometers. Depending on the definition of MetS, the studies from Korea showed a lower prevalence of MetS compared to other studies. Table 1 summarizes the characteristics of each study.

**Figure 2.**
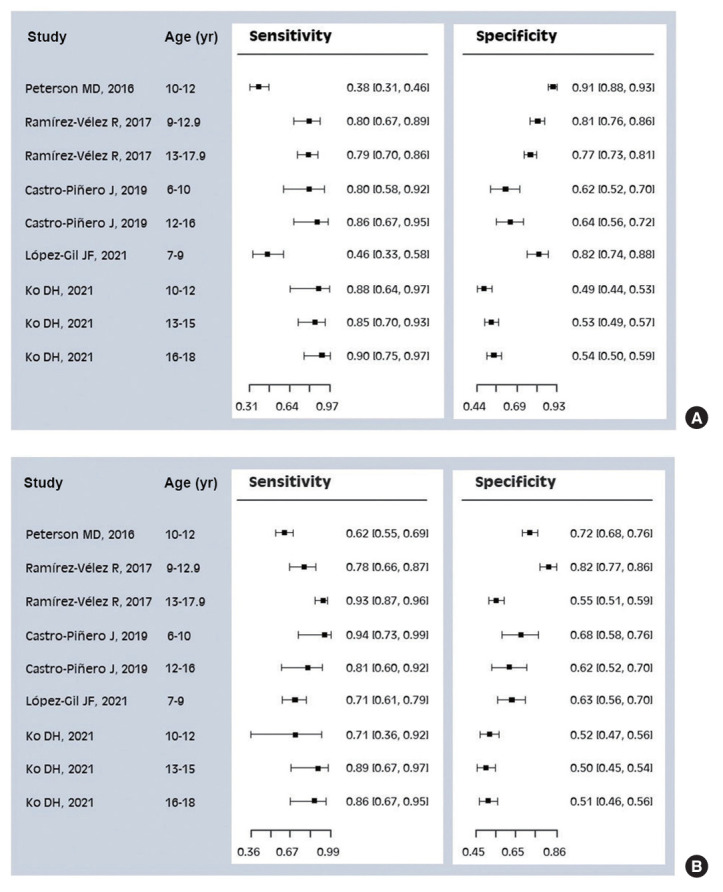
Forest plot of sensitivity and specificity for metabolic health risk evaluated by handgrip strength. The sensitivity and specificity values are stratified by boys (A) and girls (B). The data are presented for each sex using 9 subsets from 5 studies.

Summary points of sensitivity and specificity for the detection of MetS risk by HGS were estimated using a bivariate model using 18 parameters derived from 5 studies. The study sample size was 3,471 boys and 3,587 girls. Supplementary Material 3 presents the characteristics of individual studies for the bivariate models. The positive LR for that threshold was highest in the Colombian study for children aged 9.0-12.9 years, and the negative LR was lowest in the Spanish study for girls aged 6-10 years. The ranges of sensitivity and specificity were 0.62 to 0.94 and 0.50 to 0.82 for girls, and 0.38 to 0.90 and 0.49 to 0.91 for boys, respectively (Figure 2). The overall summary values for sensitivity and specificity were 0.80 and 0.66, respectively, and the summary AUC was 0.79. The sensitivity and specificity values by sex were 0.78 and 0.70 for boys and 0.82 and 0.62 for girls, respectively. The corresponding values were estimated to be 0.68 and 0.69 for children and 0.82 and 0.65 for adolescents, respectively. All *I*^2^ values for heterogeneity were found to be lower than 50% (Table 2).

**Figure 3.**
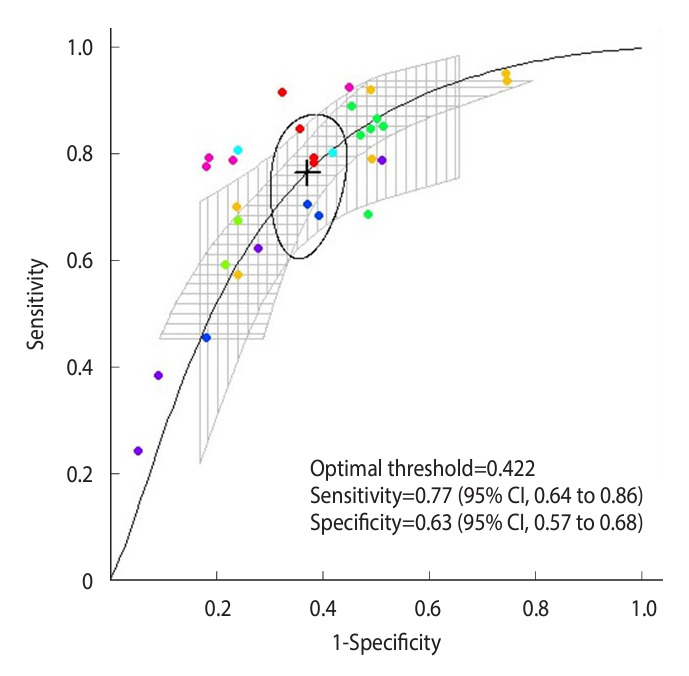
Multiple thresholds summary receiver operating characteristic curve for handgrip strength to assess metabolic health risk. Multiple thresholds summary receiver operating characteristic curves were obtained from multiple thresholds using all available data (n=32 from the 8 studies). Circles represent the sensitivity and specificity of individual data, and data derived from the same study are shown in the same color. The cross mark indicates the optimal threshold that is surrounded by its 95% confidence region. Vertical hatching corresponds to pointwise confidence intervals (CIs) for sensitivity values relative to their specificity, and horizontal hatching corresponds to pointwise CIs for specificity values relative to their sensitivity. Information on sensitivity and specificity at the optimal threshold is shown in the figure.

**Figure 4.**
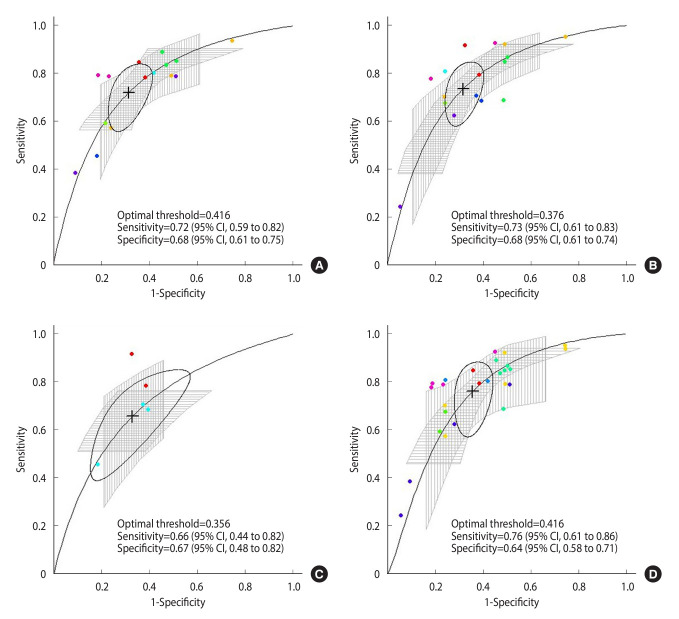
Subgroup analysis for multiple thresholds summary receiver operating characteristic curves for handgrip strength to assess metabolic health risk. Multiple thresholds summary receiver operating characteristic curves were obtained from multiple thresholds using all available data for boys (A), girls (B), children (C), and adolescents (D). Circles represent the sensitivity and specificity of individual data, and data derived from the same study are shown in the same color. The cross mark indicates the optimal threshold. Information on sensitivity and specificity for each optimal threshold is shown in each graph. CI, confidence interval.

Regarding the meta-analysis of multiple thresholds, across the 8 studies, the thresholds ranged from 0.33 to 0.58 for boys and 0.28 to 0.47 for girls (Supplementary Material 4). The optimal threshold was identified as 0.422, and the accuracy at that point showed a sensitivity of 0.77 and a specificity of 0.63 (Figure 3). In the subgroup analysis, the optimal threshold according to sex was 0.416 for boys and 0.376 for girls, and the sensitivity and specificity values of the optimal threshold were 0.72 and 0.68 for boys and 0.73 and 0.68 for girls. The optimal threshold by age group was estimated to be 0.356 in children and 0.416 in adolescents. The sensitivity and specificity of the corresponding values were 0.66 and 0.67 for children and 0.76 and 0.64 for adolescents (Figure 4). In the sensitivity analysis, it was observed that the CIs for sensitivity widened after the exclusion of a study from the United States for its differences in measurement tools (Supplementary Material 5).

## DISCUSSION

This meta-analysis aimed to evaluate the diagnostic accuracy of HGS for MetS risk systematically and propose optimal thresholds for HGS, focusing on children and adolescents. The optimal threshold for HGS (defined as relative HGS by adjusting for body mass) to detect MetS risk was found to be 0.416 for boys and 0.376 for girls. Regarding diagnostic accuracy, the summary sensitivity was higher than the summary specificity across both sexes. The summary AUC value was 0.79, showing that HGS could detect MetS risk at a fair level. It was at a similar level in the subgroups divided by sex or age group.

A systematic review conducted by de Lima et al. [9] suggested a beneficial effect of muscle strength on MetS risk in children and adolescents, but no quantitative meta-analysis has been conducted because of the heterogeneity among studies regarding MetS definitions and muscle strength measurements. Another study comprehensively reviewed the effects of muscle strength, power, and endurance relative to health-related outcomes in children and adolescents aged 4 years to 17 years. Based on data from 4 studies, the median AUC for detecting MetS risk based on muscle strength measured by HGS was summarized as 0.80 [10]. Additionally, a meta-analysis in adults reported an inverse relationship between HGS and MetS in a dose-response manner (odds ratio, 0.68 per 0.1 unit of HGS/kg; 95% CI, 0.62 to 0.75) [11]. Some studies have demonstrated an association between HGS and metabolic risk in terms of prospective findings [20,22], but not all [23]. Beyond this, our study provides practical information for detecting high-risk groups to encourage muscle strength-related activities that may reduce potential MetS risk in children and adolescents, but several issues remain to be addressed.

MetS is a condition accompanied by unhealthy metabolic factors such as obesity, high blood pressure, high fasting blood sugar, high triglyceride levels, and low levels of high-density lipoprotein (HDL) cholesterol. However, the heterogeneity of studies regarding MetS definitions for children and adolescents has led to a wide range of prevalence rates [24]. Some studies calculated cMetS and defined participants with ≥ 1 standard deviation (SD) as at risk of MetS [8,20,21]. Both the components of MetS and the criteria for those components have varied across studies. Adolescence brings physiological changes that can alter metabolic markers and potentially affect how MetS occurs [9]. Meanwhile, another study showed inconsistent results when comparing HGS and MetS health risks by sex [25,26]. How MetS is determined in children and adolescents overlooks developmental differences based on age and sex, and the IDF definition states that MetS cannot be diagnosed in children under 10 years of age [27]. One cohort study tracked individual cMetS from age 3 through adolescence and found a stable pattern [28]. Studies conducted in Chile and Spain have also assessed MetS risk using cMetS in children under 10 years of age [20,21]. Susceptibility to MetS can be observed even in young children. Accordingly, from a public health perspective, establishing a consistent definition of MetS is necessary to identify appropriate indicators associated with MetS risk, including in younger individuals.

Physical fitness can be assessed using a variety of indicators, including HGS. HGS is a simple and inexpensive assessment tool that correlates to overall muscle strength [29]. HGS has been reported as having higher discriminatory power for detecting MetS risk than other indicators [10]. To control the confounding effect, all studies included in the meta-analysis used relative HGS. One study found that the relative HGS value had a higher AUC than the absolute HGS value for detecting MetS or cardiometabolic risk [6]. Nevertheless, variation in HGS measurements may arise from diverse factors such as geography, ethnicity, and age, contributing to heterogeneity. The most pressing issue is the lack of a standard measurement method for HGS. To measure HGS, hydraulic or spring-type dynamometers are mentioned frequently in the literature. The Jamar hydraulic dynamometer used in one United States study is generally considered to be the standard measurement tool [30]. Both types of instruments are reported to be very reliable for measuring HGS in children [31]. The use of spring-type TKK dynamometers (Takei, Tokyo, Japan) is increasingly common due to their convenience. Though a strong correlation was shown in one study when HGS was measured in children with a Jamar hydraulic dynamometer [32], the study did not explain sufficiently clearly how the measurement tools could be compared. Therefore, further research would be required to address heterogeneity due to differences in measurement tools.

Engaging in regular physical activity is crucial for managing MetS health risks. Some research has indicated that physical activity not only mitigates health issues related to MetS, including adiposity and inflammation, but also strengthens physical fitness, bone health, and cognitive performance [33,34]. School-based intervention studies have reported that physical activity is associated with improvements in physical fitness, muscle strength, and cardiorespiratory fitness [35]. The World Health Organization has published guidelines on physical activity for children and adolescents [36]. Despite these recommendations, a significant proportion of adolescents worldwide fail to meet these guidelines, with inactivity rates alarmingly high in certain countries [37-40]. The COVID-19 pandemic has exacerbated the issue [41], leading to higher rates of obesity and MetS among young people [42]. A pressing need has emerged for the development of school-focused and community-focused strategies and policies to encourage active participation in physical activities.

Caution is needed in interpreting the results. First, due to a lack of studies that considered children of diverse racial groups, the current study was conducted using limited data and is formally incomplete. As a result, how the results may be generalized and applied to the general pediatric population is similarly limited. Additionally, because we only considered publications in English, not all relevant studies may have been included. Finally, the study’s results may also have been affected by our estimation of values for information that was not specified. Nevertheless, our study is the first to propose an optimal threshold for HGS associated with MetS through a meta-analysis. Despite the limited number of included studies, optimal thresholds were suggested according to sex and age group. This may help identify children with low HGS and determine which children may need intervention for muscle strengthening due to their poor metabolic health. Additional research is needed to validate our findings.

## CONCLUSION

In summary, we provided information regarding the performance of screening tools for HGS assessment of poor metabolic health in children and adolescents. Additionally, by proposing optimal thresholds of HGS through the multiple thresholds model, we provided practical information that can be used in public healthcare or primary clinical practice. However, for this proposal to be valid, establishing a standardized MetS definition and HGS measurement method must be prioritized.

## Figures and Tables

**Figure f5-epih-46-e2024047:**
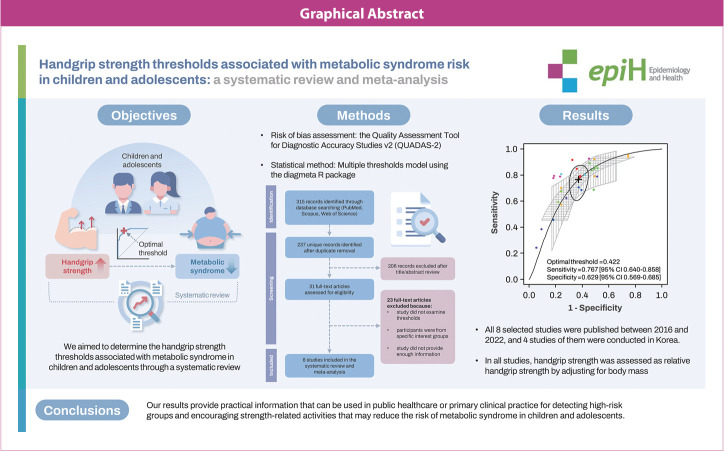


**Table 1.** Characteristics of included studies

Study	Country	Data	Age, range (yr)	n	Definition of MetS	MetS (%)	Definition of HGS	HGS measuring tools	No. of thresholds
Peterson et al., 2016 [7]	USA	Cardiovascular Health Intervention Program	10-12	1,326 (boys=630, girls=696)	Cardiometabolic risk score ≥75th percentile, computed using the sum of the standardized scores of body fat percentage, fasting glucose, BP, TG, and HDL-C	25.6	Average of HGS values of dominant hand divided by body mass	Jamar hydraulic handgrip dynamometer	4
Ramírez-Vélez et al., 2017 [8]	Colombia	FUPRECOL study	9-17.9	1,950 (boys=859, girls=1,091)	Cardiometabolic risk score >1.0 SD, computed using the sum of the age and sex standardized scores of WC, TG, HDL-C, glucose, SBP, and DBP	15.9	Average of the maximum HGS values for each hand divided by body mass	Takei spring-type dynamometer (TKK 540^®^)	4
Ko et al., 2021 [15]	Korea	KNHANES 2014-2018	10-18	2,819 (boys=1,527, girls=1,292)	MetS was diagnosed when 3 or more of the following criteria were fulfilled: WC ≥90th percentile, BP ≥90th percentile, TG ≥110 mg/dL, HDL-C ≤40 mg/dL, fasting glucose ≥110 mg/dL, and prescription of BP medication, hyperlipidemia medication, or diabetes medication	4.7	Maximum values of HGS divided by body mass	Takei spring-type dynamometer (TKK 5401)	6
Choi, 2021 [16]	Korea	KNHANES 2014-2018	10-18	2,802 (boys=1,491, girls=1,311)	International Diabetes Federation^1^	2.5	Sum of the maximum values of HGS for each hand divided by body mass	Takei spring-type dynamometer (TKK 5401)	6
Lee et al., 2022 [17]	Korea	KNHANES 2014-2017	10-18	2,303 (boys=1,226, girls=1,077)	International Diabetes Federation^1^	2.8	Maximum values of HGS divided by body mass	Takei spring-type dynamometer (TKK 5401)	2
Jung et al., 2022 [18]	Korea	KNHANES 2014-2018	10-18	2,797 (boys=1,487, girls=1,310)	International Diabetes Federation^1^	2.0	The sum of the maximum values of HGS for each hand divided by body mass	Takei spring-type dynamometer (TKK 5401)	2
Castro-Piñero et al., 2019 [20]	Spain	UP&DOWN study	6-16	511 (boys=270, girls=241)	CVD risk score >1.0 SD, computed using the sum of the standardized score of 2 skinfolds, SBP, insulin, glucose, TG, and total cholesterol/HDL-C	15.7	Average of the maximum HGS values for each hand divided by body mass	Takei spring-type dynamometer (TKK 5101 Grip D)	4
López-Gil et al., 2021 [21]	Chile	Growth and Obesity Chilean Cohort Study	7-9	452 (boys=185, girls=267)	(Equation 1) Cardiometabolic risk score >1.0 SD, computed using the sum of WHtR-z, insulin-z, triglycerides-z, HDL-z, and glycemia-z	(Eq1) 31.0, (Eq2) 31.6	Average of the maximum HGS values for each hand divided by body mass	Smedley spring-type dynamometer (Baseline 12-0286^®^)	4
					(Equation 2) Cardiometabolic risk score >1.0 SD, computed using the sum of WC-z, insulin-z, triglycerides-z, HDL-z, and glycemia-z				

MetS, metabolic syndrome; HGS, handgrip strength; WC, waist circumference; TG, triglyceride; HDL-C, high-density lipoprotein cholesterol; BP, blood pressure; SBP, systolic blood pressure; DBP, diastolic blood pressure; CVD, cardiovascular disease; SD, standard deviation; WHtR, waist to height ratio; KNHANES, Korea National Health and Nutrition Examination Survey.

1 Subjects were classified as having MetS if they had a high WC (age- and sex-specific WC ≥90th percentile for 10-15 years of age, WC ≥90 cm for boys and ≥80 cm for girls with 16-18 years of age) and 2 or more of the indicated metabolic risk factors (BP ≥130/85 mmHg, glucose ≥100 mg/dL, TG ≥150 mg/dL, HDL-C <40 mg/dL for individuals 10-15 years of age and boys 16-18 years of age, and HDL-C <50 mg/dL for girls 16-18 years of age).

**Table 2.** Systematic estimation of sensitivity and specificity for metabolic health risk evaluated by HGS^1^

Variables	No. parameters (no. studies)	Sensitivity (95% CI)	Specificity (95% CI)	AUC	*I*^2^ ^2^
Total	18 (5)	0.80 (0.72, 0.86)	0.66 (0.59, 0.73)	0.79	32.2
Sex					
Boys	9 (5)	0.78 (0.64, 0.87)	0.70 (0.58, 0.80)	0.80	45.2
Girls	9 (5)	0.82 (0.72, 0.88)	0.62 (0.54, 0.69)	0.77	48.4
Age group					
Children	4 (2)	0.68 (0.50, 0.81)	0.69 (0.58, 0.78)	0.73	0.0
Adolescents	14 (3)	0.82 (0.74, 0.88)	0.65 (0.56, 0.73)	0.80	43.0

HGS, handgrip strength; CI, confidence interval; AUC, area under the curve.

1 The analysis is based on the bivariate model (using 1 pair of sensitivity and specificity values by sex and age group) from the 5 studies.

2 Zhou and Dendukuri approach.

## Supplementary materials

Supplementary Material 1. Search terms related to handgrip strength and metabolic health in children and adolescents

Supplementary Material 2. Risk of bias assessment based on the quality assessment of diagnostic accuracy studies (QUADAS-2) for included studies

Supplementary Material 3. Characteristics of included studies for bivariate model

Supplementary Material 4. Characteristics of included studies for multiple thresholds model

Supplementary Material 5. Sensitivity analysis of multiple thresholds summary receiver operating characteristic curve for HGS to detecting metabolic health risk
