# Supplementary material for: Handgrip strength thresholds associated with metabolic syndrome risk in children and adolescents: a systematic review and meta-analysis
Source: Epidemiol Health. 2024 Apr 24;46:e2024047. doi: 10.4178/epih.e2024047 (PMC11573490; doi:10.4178/epih.e2024047)
Supplement: Supplementary Material 1. — Search terms related to handgrip strength and metabolic health in children and adolescents [file epih-46-e2024047-Supplementary-1.docx]

**Supplementary Material 1**. Search terms related to handgrip strength and metabolic health in children and adolescents

| PubMed | ("hand strength"[All Fields] OR "handgrip"[All Fields] OR "handgrip strength"[All Fields] OR "holding power"[All Fields] OR "hand strengths"[All Fields] OR "strength hand"[All Fields] OR "strengths hand"[All Fields] OR "grip"[All Fields] OR "grips"[All Fields] OR "grasp"[All Fields] OR "grasps"[All Fields] OR "hand grip"[All Fields] OR "grip power"[All Fields] OR "grip strength"[All Fields] OR "muscle-strength"[All Fields] OR "muscular fitness"[All Fields] OR "muscle strength dynamometer"[All Fields] OR "grip dynamometer"[All Fields] OR "muscle weakness"[All Fields] OR "muscular strength"[All Fields] OR "hand/physiology"[All Fields] OR "strength muscle"[All Fields] OR "muscle force"[All Fields] OR "muscle-strength"[All Fields]) AND ("metabolic syndrome"[All Fields] OR "metabolic syndromes"[All Fields] OR "syndrome metabolic"[All Fields] OR "metabolic syndrome x"[All Fields] OR "insulin resistance syndrome x"[All Fields] OR "syndrome x metabolic"[All Fields] OR "syndrome x insulin resistance"[All Fields] OR "metabolic x syndrome"[All Fields] OR "syndrome metabolic x"[All Fields] OR "x syndrome metabolic"[All Fields] OR "dysmetabolic syndrome x"[All Fields] OR "reaven syndrome x"[All Fields] OR "syndrome x reaven"[All Fields] OR "cardiovascular syndrome metabolic"[All Fields] OR "cardiometabolic syndrome"[All Fields] OR "cardiometabolic syndromes"[All Fields] OR "syndrome cardiometabolic"[All Fields] OR "metabolic diseases"[All Fields] OR "metabolic disease"[All Fields] OR "metabolic abnormality"[All Fields] OR "metabolic abnormalities"[All Fields] OR "metabolic disorder"[All Fields] OR "insulin-resistance"[All Fields] OR "syndrome metabolic cardiovascular"[All Fields] OR "metabolic cardiovascular syndrome"[All Fields] OR "cardiovascular syndrome metabolic"[All Fields] OR "syndrome metabolic cardiovascular"[All Fields]) AND ("sensitive"[All Fields] OR "sensitively"[All Fields] OR "sensitives"[All Fields] OR "sensitivities"[All Fields] OR "Sensitivity and Specificity"[MeSH Terms] OR ("sensitivity"[All Fields] AND "specificity"[All Fields]) OR "Sensitivity and Specificity"[All Fields] OR "sensitivity"[All Fields] OR ("Sensitivity and Specificity"[MeSH Terms] OR ("sensitivity"[All Fields] AND "specificity"[All Fields]) OR "Sensitivity and Specificity"[All Fields] OR "specificity"[All Fields] OR "specific"[All Fields] OR "specifically"[All Fields] OR "specification"[All Fields] OR "specifications"[All Fields] OR "specificities"[All Fields] OR "specifics"[All Fields] OR "specifities"[All Fields] OR "specifity"[All Fields]) OR "Sensitivity and Specificity"[All Fields] OR "Predictive value of tests"[All Fields] OR "ROC curve"[All Fields] OR "roc analyses"[All Fields] OR "diagnostic accuracy"[All Fields] OR "ROC analysis"[All Fields] OR "receiver operating characteristic"[All Fields] OR ("cutoff"[All Fields] OR "cutoffs"[All Fields]) OR ("threshold"[All Fields] OR "thresholds"[All Fields])) AND ("child"[MeSH Terms] OR "child"[All Fields] OR "children"[All Fields] OR "child s"[All Fields] OR "children s"[All Fields] OR "childrens"[All Fields] OR "childs"[All Fields] OR ("child"[MeSH Terms] OR "child"[All Fields] OR "children"[All Fields] OR "child s"[All Fields] OR "children s"[All Fields] OR "childrens"[All Fields] OR "childs"[All Fields]) OR ("paediatrics"[All Fields] OR "pediatrics"[MeSH Terms] OR "pediatrics"[All Fields] OR "paediatric"[All Fields] OR "pediatric"[All Fields]) OR ("adolescences"[All Fields] OR "adolescency"[All Fields] OR "adolescent"[MeSH Terms] OR "adolescent"[All Fields] OR "adolescence"[All Fields] OR "adolescents"[All Fields] OR "adolescent s"[All Fields]) OR ("adolescences"[All Fields] OR "adolescency"[All Fields] OR "adolescent"[MeSH Terms] OR "adolescent"[All Fields] OR "adolescence"[All Fields] OR "adolescents"[All Fields] OR "adolescent s"[All Fields]) OR "adolescent health"[All Fields] OR "child health"[All Fields]) |
| --- | --- |
| Web of Science | (((TS=((metabolic syndrome OR metabolic syndromes OR syndrome metabolic OR metabolic syndrome x OR insulin resistance syndrome x OR syndrome x metabolic OR syndrome x insulin resistance OR metabolic x syndrome OR syndrome metabolic x OR x syndrome metabolic OR dysmetabolic syndrome x OR reuven syndrome x OR syndrome x reuven OR cardiovascular syndrome metabolic OR cardiometabolic syndrome OR cardiometabolic syndromes OR syndrome cardiometabolic OR metabolic diseases OR metabolic disease OR metabolic abnormality OR metabolic abnormalities OR metabolic disorder OR insulin-resistance OR syndrome metabolic cardiovascular OR metabolic cardiovascular syndrome OR cardiovascular syndrome, metabolic OR syndrome, metabolic cardiovascular))) AND TS=((hand strength OR handgrip OR handgrip strength OR holding power OR hand strengths OR strength hand OR strengths hand OR grip OR grips OR grasp OR grasps OR hand grip OR grip power OR grip strength OR muscle strength OR muscular fitness OR muscle strength dynamometer OR grip dynamometer OR muscle weakness OR muscular strength OR hand/physiology OR muscle force OR muscle-strength))) AND TS=((Sensitivity OR Specificity OR Sensitivity and Specificity OR Predictive value of tests OR ROC curve OR roc analyses OR diagnostic accuracy OR ROC analysis OR receiver operating characteristic OR cutoff OR thresholds))) AND TS=((child OR children OR pediatric OR adolescent OR adolescents OR adolescent health OR child health)) |
| SCOPUS | TITLE-ABS-KEY ( ( "muscle-strength" OR "muscle force" OR "strength, muscle" OR "hand/physiology" OR "muscular strength" OR "muscle weakness" OR "grip dynamometer" OR "muscle strength dynamometer" OR "muscular fitness" OR "muscle strength" OR "grip strength" OR "grip power" OR "hand grip" OR "grasps" OR "grasp" OR "grips" OR "grip" OR "strengths hand" OR "strength hand" OR "hand strengths" OR "holding power" OR "handgrip strength" OR "handgrip" OR "hand strength" ) AND ( "syndrome, metabolic cardiovascular" OR "cardiovascular syndrome, metabolic" OR "metabolic cardiovascular syndrome" OR "syndrome metabolic cardiovascular" OR "insulin-resistance" OR "metabolic disorder" OR "metabolic abnormalities" OR "metabolic abnormality" OR "metabolic disease" OR "metabolic diseases" OR "syndrome cardiometabolic" OR "cardiometabolic syndromes" OR "cardiometabolic syndrome" OR "cardiovascular syndrome metabolic" OR "syndrome x reaven" OR "reaven syndrome x" OR "dysmetabolic syndrome x" OR "x syndrome metabolic" OR "syndrome metabolic x" OR "metabolic x syndrome" OR "syndrome x insulin resistance" OR "syndrome x metabolic" OR "insulin resistance syndrome x" OR "metabolic syndrome x" OR "syndrome metabolic" OR "metabolic syndromes" OR "metabolic syndrome" ) AND ( thresholds OR cutoff OR "receiver operating characteristic" OR "ROC analysis" OR "diagnostic accuracy" OR "roc analyses" OR "ROC curve" OR "Predictive value of tests" OR "Sensitivity and Specificity" OR specificity OR sensitivity ) AND ( "child health" OR "adolescent health" OR adolescents OR adolescent OR pediatric OR children OR child ) ) |
