# Supplementary material for: Handgrip strength thresholds associated with metabolic syndrome risk in children and adolescents: a systematic review and meta-analysis
Source: Epidemiol Health. 2024 Apr 24;46:e2024047. doi: 10.4178/epih.e2024047 (PMC11573490; doi:10.4178/epih.e2024047)
Supplement: Supplementary Material 2. — Risk of bias assessment based on the quality assessment of diagnostic accuracy studies (QUADAS-2) for included studies [file epih-46-e2024047-Supplementary-2.docx]

**Supplementary Material 2**. Risk of bias assessment based on the quality assessment of diagnostic accuracy studies (QUADAS-2) for included studies
