# Supplementary material for: Handgrip strength thresholds associated with metabolic syndrome risk in children and adolescents: a systematic review and meta-analysis
Source: Epidemiol Health. 2024 Apr 24;46:e2024047. doi: 10.4178/epih.e2024047 (PMC11573490; doi:10.4178/epih.e2024047)
Supplement: Supplementary Material 3. — Characteristics of included studies for bivariate model [file epih-46-e2024047-Supplementary-3.docx]

**Supplementary Material 3**. Characteristics of included studies for bivariate model

| **Study.no.** | **First author** | **Year** | **Country** | **Age (years)** | **Gender** | **Size** | **MetS (%)** | **Cutoff of HGS** | **TP** | **FP** | **FN** | **TN** | **Sensitivity (95% CI)** | **Specificity**  **(95% CI)** | **posLR**  **(95% CI)** | **negLR**  **(95% CI)** | **DOR**  **(95% CI)** |
| --- | --- | --- | --- | --- | --- | --- | --- | --- | --- | --- | --- | --- | --- | --- | --- | --- | --- |
| [7] | Peterson | 2016 | USA | 10-12 | Boys | 630 | 24.4 | 0.33 | 59 | 44 | 95 | 432 | 0.38 (0.31, 0.46) | 0.91 (0.88, 0.93) | 4.14 (2.93, 5.86) | 0.68 (0.6, 0.77) | 6.1 (3.89, 9.55) |
| [7] | Peterson | 2016 | USA | 10-12 | Girls | 696 | 26.7 | 0.36 | 116 | 142 | 70 | 368 | 0.62 (0.55, 0.69) | 0.72 (0.68, 0.76) | 2.24 (1.87, 2.68) | 0.52 (0.43, 0.63) | 4.29 (3.01, 6.12) |
| [8] | Ramirez-Velez R | 2017 | Colombia | 9–12.9 | Boys | 313 | 16.0 | 0.376 | 40 | 49 | 10 | 214 | 0.8 (0.67, 0.89) | 0.81 (0.76, 0.86) | 4.29 (3.22, 5.73) | 0.25 (0.14, 0.43) | 17.47 (8.18, 37.33) |
| [8] | Ramirez-Velez R | 2017 | Colombia | 9–12.9 | Girls | 378 | 15.9 | 0.359 | 47 | 58 | 13 | 260 | 0.78 (0.66, 0.87) | 0.82 (0.77, 0.86) | 4.29 (3.28, 5.62) | 0.27 (0.16, 0.43) | 16.21 (8.24, 31.89) |
| [8] | Ramirez-Velez R | 2017 | Colombia | 13–17.9 | Boys | 546 | 15.9 | 0.447 | 69 | 106 | 18 | 353 | 0.79 (0.7, 0.86) | 0.77 (0.73, 0.81) | 3.43 (2.82, 4.19) | 0.27 (0.18, 0.41) | 12.77 (7.28, 22.4) |
| [8] | Ramirez-Velez R | 2017 | Colombia | 13–17.9 | Girls | 713 | 16.0 | 0.44 | 106 | 269 | 8 | 330 | 0.93 (0.87, 0.96) | 0.55 (0.51, 0.59) | 2.07 (1.87, 2.29) | 0.13 (0.07, 0.25) | 16.25 (7.78, 33.95) |
| [16] | Castro-Pinero J | 2019 | Spain | 6-10 | Boys | 127 | 15.7 | 0.367 | 16 | 41 | 4 | 66 | 0.8 (0.58, 0.92) | 0.62 (0.52, 0.7) | 2.09 (1.51, 2.89) | 0.32 (0.13, 0.79) | 6.44 (2.01, 20.6) |
| [16] | Castro-Pinero J | 2019 | Spain | 6-10 | Girls | 110 | 15.5 | 0.306 | 16 | 30 | 1 | 63 | 0.94 (0.73, 0.99) | 0.68 (0.58, 0.76) | 2.92 (2.12, 4.01) | 0.09 (0.01, 0.58) | 33.6 (4.25, 265.35) |
| [16] | Castro-Pinero J | 2019 | Spain | 12-16 | Boys | 143 | 15.4 | 0.473 | 19 | 43 | 3 | 78 | 0.86 (0.67, 0.95) | 0.64 (0.56, 0.72) | 2.43 (1.82, 3.25) | 0.21 (0.07, 0.61) | 11.49 (3.22, 41.04) |
| [16] | Castro-Pinero J | 2019 | Spain | 12-16 | Girls | 131 | 16.0 | 0.423 | 17 | 42 | 4 | 68 | 0.81 (0.6, 0.92) | 0.62 (0.52, 0.7) | 2.12 (1.55, 2.91) | 0.31 (0.13, 0.75) | 6.88 (2.17, 21.84) |
| [17] | Lopez-Gil JF | 2021 | Chile | 7-9 | Boys | 185 | 31 | 0.33 | 26 | 23 | 31 | 105 | 0.46 (0.33, 0.58) | 0.82 (0.74, 0.88) | 2.54 (1.59, 4.05) | 0.66 (0.52, 0.85) | 3.83 (1.92, 7.63) |
| [17] | Lopez-Gil JF | 2021 | Chile | 7-9 | Girls | 267 | 32 | 0.4 | 61 | 67 | 25 | 114 | 0.71 (0.61, 0.79) | 0.63 (0.56, 0.7) | 1.92 (1.52, 2.42) | 0.46 (0.33, 0.65) | 4.15 (2.38, 7.23) |
| [18] | Ko DH | 2021 | Korea | 10-12 | Boys | 496 | 3.2 | 0.349 | 14 | 247 | 2 | 233 | 0.88 (0.64, 0.97) | 0.49 (0.44, 0.53) | 1.7 (1.39, 2.09) | 0.26 (0.07, 0.94) | 6.6 (1.48, 29.37) |
| [18] | Ko DH | 2021 | Korea | 10-12 | Girls | 424 | 1.6 | 0.373 | 5 | 205 | 2 | 218 | 0.71 (0.36, 0.92) | 0.52 (0.47, 0.56) | 1.47 (0.91, 2.38) | 0.55 (0.17, 1.8) | 2.66 (0.51, 13.86) |
| [18] | Ko DH | 2021 | Korea | 13-15 | Boys | 550 | 7.1 | 0.466 | 33 | 241 | 6 | 270 | 0.85 (0.7, 0.93) | 0.53 (0.49, 0.57) | 1.79 (1.53, 2.11) | 0.29 (0.14, 0.61) | 6.16 (2.54, 14.96) |
| [18] | Ko DH | 2021 | Korea | 13-15 | Girls | 442 | 3.9 | 0.383 | 16 | 222 | 2 | 220 | 0.89 (0.67, 0.97) | 0.5 (0.45, 0.54) | 1.77 (1.47, 2.14) | 0.22 (0.06, 0.83) | 7.93 (1.8, 34.89) |
| [18] | Ko DH | 2021 | Korea | 16-18 | Boys | 481 | 6.4 | 0.485 | 28 | 205 | 3 | 245 | 0.9 (0.75, 0.97) | 0.54 (0.5, 0.59) | 1.98 (1.7, 2.31) | 0.18 (0.06, 0.52) | 11.15 (3.34, 37.22) |
| [18] | Ko DH | 2021 | Korea | 16-18 | Girls | 426 | 4.9 | 0.382 | 19 | 209 | 3 | 217 | 0.86 (0.67, 0.95) | 0.51 (0.46, 0.56) | 1.76 (1.45, 2.13) | 0.27 (0.09, 0.77) | 6.58 (1.92, 22.55) |

MetS; metabolic syndrome, HGS; handgrip strength, TP; true positive, FP; false positive; FN, false negative; TN, true negative; posLR, positive likelihood ratio; negLR, negative likelihood ratio; DOR, diagnostic odds ratio.
