# Supplementary material for: Handgrip strength thresholds associated with metabolic syndrome risk in children and adolescents: a systematic review and meta-analysis
Source: Epidemiol Health. 2024 Apr 24;46:e2024047. doi: 10.4178/epih.e2024047 (PMC11573490; doi:10.4178/epih.e2024047)
Supplement: Supplementary Material 4. — Characteristics of included studies for multiple thresholds model [file epih-46-e2024047-Supplementary-4.docx]

**Supplementary Material 4**. Characteristics of included studies for multiple thresholds model

| **Study.no.** | **First author** | **Year** | **Country** | **Age-group** | **Gender** | **Size** | **MetS (%)** | **Cutoff of HGS** | **TP** | **FP** | **FN** | **TN** |
| --- | --- | --- | --- | --- | --- | --- | --- | --- | --- | --- | --- | --- |
| [7] | Peterson | 2016 | USA | 10-12 years | Boys | 630 | 24.4 | 0.33 | 95 | 432 | 59 | 44 |
| [7] | Peterson | 2016 | USA | 10-12 years | Boys | 630 | 24.4 | 0.45 | 32 | 233 | 122 | 243 |
| [7] | Peterson | 2016 | USA | 10-12 years | Girls | 696 | 26.7 | 0.36 | 70 | 368 | 116 | 142 |
| [7] | Peterson | 2016 | USA | 10-12 years | Girls | 696 | 26.7 | 0.28 | 141 | 483 | 45 | 27 |
| [8] | Ramirez-Velez R | 2017 | Colombia | 9–12.9 years | Boys | 313 | 16.0 | 0.376 | 10 | 214 | 40 | 49 |
| [8] | Ramirez-Velez R | 2017 | Colombia | 9–12.9 years | Girls | 378 | 15.9 | 0.359 | 13 | 260 | 47 | 58 |
| [8] | Ramirez-Velez R | 2017 | Colombia | 13–17.9 years | Boys | 546 | 15.9 | 0.447 | 18 | 353 | 69 | 106 |
| [8] | Ramirez-Velez R | 2017 | Colombia | 13–17.9 years | Girls | 713 | 16.0 | 0.44 | 8 | 330 | 106 | 269 |
| [16] | Castro-Pinero J | 2019 | Spain | 6-10 years | Boys | 127 | 15.7 | 0.367 | 4 | 66 | 16 | 41 |
| [16] | Castro-Pinero J | 2019 | Spain | 6-10 years | Girls | 110 | 15.5 | 0.306 | 1 | 63 | 16 | 30 |
| [16] | Castro-Pinero J | 2019 | Spain | 12-16 years | Boys | 143 | 15.4 | 0.473 | 3 | 78 | 19 | 43 |
| [16] | Castro-Pinero J | 2019 | Spain | 12-16 years | Girls | 131 | 16.0 | 0.423 | 4 | 68 | 17 | 42 |
| [17] | Lopez-Gil JF | 2021 | Chile | 7-9 years | Boys | 185 | 31 | 0.33 | 31 | 105 | 26 | 23 |
| [17] | Lopez-Gil JF | 2021 | Chile | 7-9 years | Girls | 267 | 31 | 0.4 | 26 | 112 | 57 | 72 |
| [17] | Lopez-Gil JF | 2021 | Chile | 7-9 years | Boys | 185 | 31 | 0.33 | 31 | 105 | 26 | 23 |
| [17] | Lopez-Gil JF | 2021 | Chile | 7-9 years | Girls | 267 | 32 | 0.4 | 25 | 114 | 61 | 67 |
| [18] | Ko DH | 2021 | Korea | 10-12 years | Boys | 496 | 3.2 | 0.349 | 2 | 233 | 14 | 247 |
| [18] | Ko DH | 2021 | Korea | 10-12 years | Girls | 424 | 1.6 | 0.373 | 2 | 218 | 5 | 205 |
| [18] | Ko DH | 2021 | Korea | 13-15 years | Boys | 550 | 7.1 | 0.466 | 6 | 270 | 33 | 241 |
| [18] | Ko DH | 2021 | Korea | 13-15 years | Girls | 442 | 3.9 | 0.383 | 2 | 220 | 16 | 222 |
| [18] | Ko DH | 2021 | Korea | 16-18 years | Boys | 481 | 6.4 | 0.485 | 3 | 245 | 28 | 205 |
| [18] | Ko DH | 2021 | Korea | 16-18 years | Girls | 426 | 4.9 | 0.382 | 3 | 217 | 19 | 209 |
| [19] | Choi EY | 2021 | Korea | 10-18 years | Boys | 1491 | 2.7 | 0.41 | 17 | 1102 | 23 | 349 |
| [19] | Choi EY | 2021 | Korea | 10-18 years | Boys | 1491 | 2.7 | 0.49 | 8 | 737 | 32 | 714 |
| [19] | Choi EY | 2021 | Korea | 10-18 years | Boys | 1491 | 2.7 | 0.58 | 2 | 370 | 38 | 1081 |
| [19] | Choi EY | 2021 | Korea | 10-18 years | Girls | 1311 | 2.4 | 0.36 | 9 | 975 | 22 | 305 |
| [19] | Choi EY | 2021 | Korea | 10-18 years | Girls | 1311 | 2.4 | 0.41 | 2 | 652 | 29 | 628 |
| [19] | Choi EY | 2021 | Korea | 10-18 years | Girls | 1311 | 2.4 | 0.47 | 1 | 327 | 30 | 953 |
| [20] | Lee CH | 2022 | Korea | 10-18 years | Boys | 1226 | 2.6 | 0.5 | 6 | 693 | 26 | 501 |
| [20] | Lee CH | 2022 | Korea | 10-18 years | Girls | 1077 | 3.1 | 0.38 | 6 | 793 | 27 | 251 |
| [21] | Jung HW | 2022 | Korea | 10-18 years | Boys | 1487 | 2.5 | 0.4 | 15 | 1133 | 22 | 317 |
| [21] | Jung HW | 2022 | Korea | 10-18 years | Girls | 1310 | 1.5 | 0.36 | 6 | 979 | 13 | 312 |

MetS; metabolic syndrome, HGS; handgrip strength, TP; true positive, FP; false positive; FN, false negative; TN, true negative.
