# Supplementary material for: Handgrip strength thresholds associated with metabolic syndrome risk in children and adolescents: a systematic review and meta-analysis
Source: Epidemiol Health. 2024 Apr 24;46:e2024047. doi: 10.4178/epih.e2024047 (PMC11573490; doi:10.4178/epih.e2024047)
Supplement: Supplementary Material 5. — Sensitivity analysis of multiple thresholds summary receiver operating characteristic curve for HGS to detecting metabolic health risk [file epih-46-e2024047-Supplementary-5.docx]

**Supplementary Material 5**. Sensitivity analysis of multiple thresholds summary receiver operating characteristic curve for HGS to detecting metabolic health risk


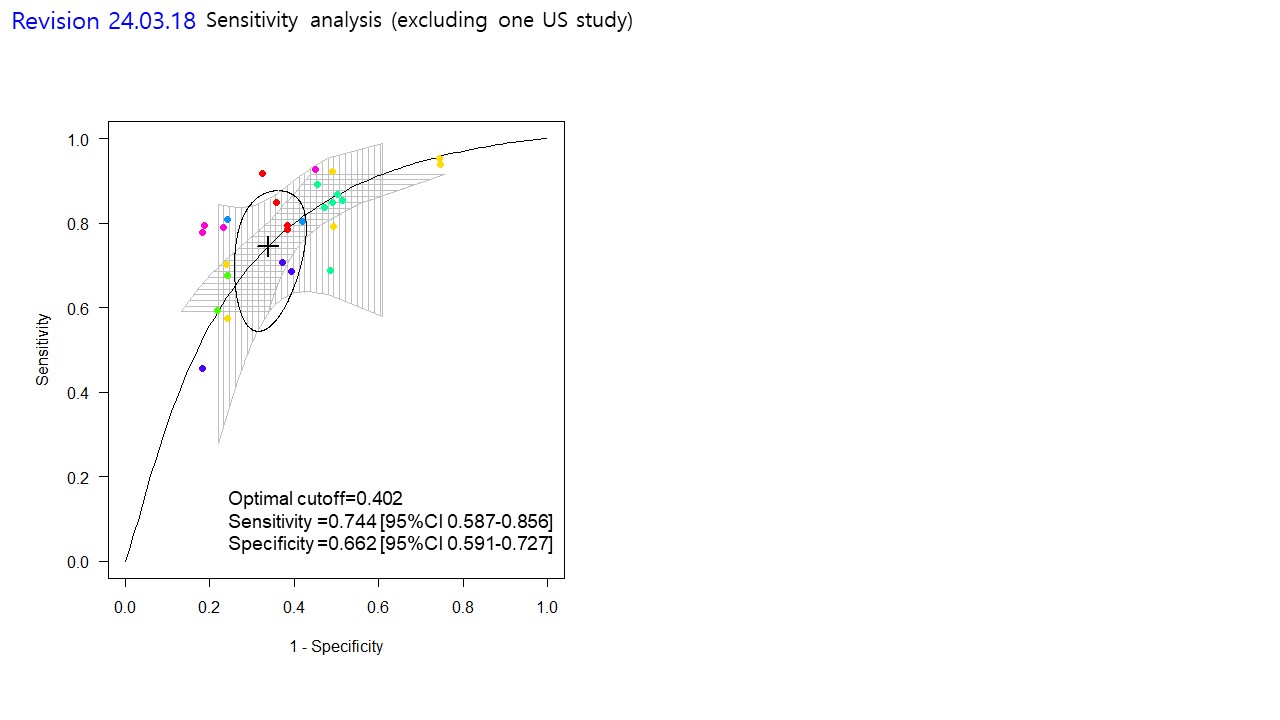


Multiple thresholds summary receiver operating characteristic curves were obtained from multiple thresholds, excluding a study from the USA due to differences in measurement tools (n=28 from the 7 studies). Circles represent the sensitivity and specificity of individual data, and data derived from the same study are shown in the same color. Cross mark indicates the optimal cutoff point that is surrounded by its 95% confidence region. Vertical hatching corresponds to pointwise confidence intervals for sensitivity, given specificity and horizontal hatching corresponds to pointwise confidence intervals for specificity given sensitivity. Information on sensitivity and specificity at optimal cutoff is shown in the figure.
